# Comparison of large language models in management advice for melanoma: Google's AI BARD, BingAI and ChatGPT

**DOI:** 10.1002/ski2.313

**Published:** 2023-11-28

**Authors:** Xin Mu, Bryan Lim, Ishith Seth, Yi Xie, Jevan Cevik, Foti Sofiadellis, David J. Hunter‐Smith, Warren M. Rozen

**Affiliations:** ^1^ Department of Plastic Surgery Peninsula Health Melbourne Victoria Australia; ^2^ Central Clinical School Monash University Melbourne Victoria Australia

## Abstract

Large language models (LLMs) are emerging artificial intelligence (AI) technology refining research and healthcare. Their use in medicine has seen numerous recent applications. One area where LLMs have shown particular promise is in the provision of medical information and guidance to practitioners. This study aims to assess three prominent LLMs—Google's AI BARD, BingAI and ChatGPT‐4 in providing management advice for melanoma by comparing their responses to current clinical guidelines and existing literature. Five questions on melanoma pathology were prompted to three LLMs. A panel of three experienced Board‐certified plastic surgeons evaluated the responses for reliability using reliability matrix (Flesch Reading Ease Score, the Flesch‐Kincaid Grade Level and the Coleman‐Liau Index), suitability (modified DISCERN score) and comparing them to existing guidelines. *t*‐Test was performed to calculate differences in mean readability and reliability scores between LLMs and *p* value <0.05 was considered statistically significant. The mean readability scores across three LLMs were same. ChatGPT exhibited superiority with a Flesch Reading Ease Score of 35.42 (±21.02), Flesch–Kincaid Grade Level of 11.98 (±4.49) and Coleman–Liau Index of 12.00 (±5.10), however all of these were insignificant (*p* > 0.05). Suitability‐wise using DISCERN score, ChatGPT 58 (±6.44) significantly (*p* = 0.04) outperformed BARD 36.2 (±34.06) and was insignificant to BingAI's 49.8 (±22.28). This study demonstrates that ChatGPT marginally outperforms BARD and BingAI in providing reliable, evidence‐based clinical advice, but they still face limitations in depth and specificity. Future research should improve LLM performance by integrating specialized databases and expert knowledge to support patient‐centred care.

1

**What is already known about this topic?**

- AI technology is being utilized to improve the screening and diagnosis of melanoma, assist clinicians in therapeutic choices and detect melanoma using deep convolutional neural networks. AI‐driven tools for melanoma diagnosis, such as Dermalyser, show promising results in decreasing the workload for dermatologists. AI‐based technologies can help improve the accuracy and efficiency of melanoma diagnosis and management.

**What does this study add?**

- This study shows that future research should enhance LLMs with specialized databases and expert insights for better patient care. This contributes to the expanding research on AI in melanoma management, emphasising LLMs' potential in clinical advice.

## 2 INTRODUCTION

Large language models (LLMs) represent a cutting‐edge application of artificial intelligence (AI) in the realm of natural language processing, with significant potential for the healthcare sector.^1^, ^2^, ^3^ By training LLMs on extensive clinical data and medical literature, it becomes possible to develop AI systems that can support doctors and other healthcare professionals in diagnosing conditions, interpreting test results and identifying suitable clinical trial opportunities for patients.^4^

Melanoma, a malignant tumour arising from melanocytes, is the most aggressive and deadly form of skin cancer.^5^ Given the growing global incidence of melanoma, there is a pressing need for precise and efficient tools to manage this formidable disease.^6^ In this context, advancements in AI, particularly in the area of LLMs, have demonstrated the potential to generate valuable insights and recommendations across various domains, offering clinicians an efficient and effective way to gather medical information and guidance.

Three LLMs that have been at the forefront of this technology include Google's AI BARD, BingAI and ChatGPT. These three models represent the most advanced LLMs at the time of conducting this study and were therefore chosen to represent this technology. This present study aims to evaluate and compare the performance of these three leading LLMs in offering evidence‐based management guidance for melanoma patients. The present study focuses on melanoma, as established guidelines are available, enabling us to make reliable comparisons with the LLMs' responses.^7^ The study's objective is to determine each model's ability to comprehend medical literature on melanoma management, extract pertinent information and generate accurate, coherent and contextually appropriate recommendations, ultimately enabling healthcare professionals to make informed decisions in their clinical practice. By leveraging the capabilities of LLMs, healthcare professionals can more effectively tackle the intricate challenges presented by melanoma, ultimately enhancing patient outcomes and propelling the field of cancer care forward. This study marks a significant stride towards realizing the full potential of AI in transforming the management of melanoma, and by extension, other types of cancer.

## 3 MATERIALS AND METHODS

### 3.1 Objective

This study aims to compare the performance of three prominent LLMs in providing evidence‐based management advice for melanoma patients: BARD, BingAI and ChatGPT. To achieve that, we will evaluate the abilities of these LLMs to comprehend medical literature, extract pertinent information and generate accurate, coherent and contextually appropriate recommendations by comparing their answers to the current clinical guidelines. The comparison will also evaluate the efficiency, reliability, readability, potential biases and ethical considerations of each LLM in the context of melanoma management. Any difference in performance highlights a potential shortcoming in an LLM's design, providing direction for future enhancements.

### 3.2 Study design

ChatGPT, BARD and BingAI were probed with melanoma‐related questions, with their responses compared to current clinical guidelines and manually‐searched literature from databases like PubMed, Scopus and Web of Science. This was to determine any high‐level evidence literature missed by the LLMs. In addition, the Likert scale responses were evaluated together in consensus by a panel of 2 plastic surgery residents, 1 registrar and 3 Specialist Plastic and Reconstructive Surgeons with over a total of 50 years' experience on the management of skin cancer (Table 1). The evaluation criteria included reliability, evaluated using DISCERN questionnaire (range 16–80, higher scores indicate greater reliability); and readability assessed using three widely acknowledged scoring systems: Flesch Reading Ease Score (range 0–100, higher scores indicate greater ease to read), Flesch–Kincaid Grade Level (range 0–12, for example a score of 8 means the text is suitable for an eighth grader to read) and a Coleman–Liau Index (no official upper or lower limits but for example, a score of 10 means a 10th grader should be able to read) (Supplementary Table 1). Despite the existence of quantitative measurements on LLM performances, we have decided to utilize a more qualitative approach as it can capture more nuanced understandings of user experience and perception that quantitative analysis may sometimes overlook. A *t*‐test was performed thereafter to assess the statistical significance of the mean reliability and readability scores. Despite the absence of gold‐standard tests, the ones discussed prior are predominantly utilized in evaluating the readability of health‐related literature.^8^, ^9^

**TABLE 1:** Overall qualitative evaluation of large language model platforms' responses by all panel members.

Criteria	ChatGPT	BingAI	Google's BARD
The large language model provides accurate answers to questions	[ ] 1—Strongly disagree	[ ] 1—Strongly disagree	[] 1—Strongly disagree
	[ ] 2—Disagree	[ ] 2—Disagree	[ ] 2—Disagree
	[ ] 3—Neither agree or disagree	[x] 3—Neither agree or disagree	[x] 3—Neither agree or disagree
	[x]4—Agree	[ ] 4—Agree	[ ] 4—Agree
	[ ] 5—Strongly agree	[ ] 5—Strongly agree	[ ] 5—Strongly agree
The large language model is reliable when generating factual information	[ ] 1—Strongly disagree	[ ] 1—Strongly disagree	[x] 1—Strongly disagree
	[ ] 2—Disagree	[ ] 2—Disagree	[ ] 2—Disagree
	[ ] 3—Neither agree or disagree	[x] 3—Neither agree or disagree	[ ] 3—Neither agree or disagree
	[x] 4—Agree	[ ] 4—Agree	[ ] 4—Agree
	[ ] 5—Strongly agree	[ ] 5—Strongly agree	[ ] 5—Strongly agree
The large language model is proficient at understanding complex questions and providing appropriate answers	[ ] 1—Strongly disagree	[ ] 1—Strongly disagree	[x] 1—Strongly disagree
	[ ] 2—Disagree	[ ] 2—Disagree	[ ] 2—Disagree
	[ ] 3—Neither agree or disagree	[x] 3—Neither agree or disagree	[ ] 3—Neither agree or disagree
	[x] 4—Agree	[ ] 4—Agree	[ ] 4—Agree
	[ ] 5—Strongly agree	[ ] 5—Strongly agree	[ ] 5—Strongly agree
The large language model provides comprehensive information when answering questions	[ ] 1—Strongly disagree	[ ] 1—Strongly disagree	[ ] 1—Strongly disagree
	[ ] 2—Disagree	[ ] 2—Disagree	[x] 2—Disagree
	[ ] 3—Neither agree or disagree	[x] 3—Neither agree or disagree	[ ] 3—Neither agree or disagree
	[x] 4—Agree	[ ] 4—Agree	[ ] 4—Agree
	[ ] 5—Strongly agree	[ ] 5—Strongly agree	[ ] 5—Strongly agree
The large language model generates content that covers all relevant aspects of a subject	[ ] 1—Strongly disagree	[ ] 1—Strongly disagree	[x] 1—Strongly disagree
	[ ] 2—Disagree	[ ] 2—Disagree	[ ] 2—Disagree
	[ ] 3—Neither agree or disagree	[x] 3—Neither agree or disagree	[ ] 3—Neither agree or disagree
	[x] 4—Agree	[ ] 4—Agree	[ ] 4—Agree
	[ ] 5—Strongly agree	[ ] 5—Strongly agree	[ ] 5—Strongly agree
The large language model is able to provide in‐depth information for a wide range of topics	[ ] 1—Strongly disagree	[ ] 1—Strongly disagree	[x] 1—Strongly disagree
	[ ] 2—Disagree	[x] 2—Disagree	[ ] 2—Disagree
	[ ] 3—Neither agree or disagree	[ ] 3—Neither agree or disagree	[ ] 3—Neither agree or disagree
	[x] 4—Agree	[ ] 4—Agree	[ ] 4—Agree
	[ ] 5—Strongly agree	[ ] 5—Strongly agree	[ ] 5—Strongly agree
The large language model is a valuable source of general knowledge	[ ] 1—Strongly disagree	[ ] 1—Strongly disagree	[ ] 1—Strongly disagree
	[ ] 2—Disagree	[x] 2—Disagree	[x] 2—Disagree
	[ ] 3—Neither agree or disagree	[ ] 3—Neither agree or disagree	[ ] 3—Neither agree or disagree
	[ ] 4—Agree	[ ] 4—Agree	[ ] 4—Agree
	[x] 5—Strongly agree	[ ] 5—Strongly agree	[ ] 5—Strongly agree
The large language model is well‐versed in a variety of subjects	[ ] 1—Strongly disagree	[ ] 1—Strongly disagree	[ ] 1—Strongly disagree
	[ ] 2—Disagree	[x] 2—Disagree	[x] 2—Disagree
	[ ] 3—Neither agree or disagree	[ ] 3—Neither agree or disagree	[ ] 3—Neither agree or disagree
	[ ] 4—Agree	[ ] 4—Agree	[ ] 4—Agree
	[x] 5—Strongly agree	[ ] 5—Strongly agree	[ ] 5—Strongly agree
The large language model can provide useful insights and perspectives on various topics	[ ] 1—Strongly disagree	[ ] 1—Strongly disagree	[ ] 1—Strongly disagree
	[ ] 2—Disagree	[x] 2—Disagree	[x] 2—Disagree
	[ ] 3—Neither agree or disagree	[ ] 3—Neither agree or disagree	[ ] 3—Neither agree or disagree
	[x] 4—Agree	[ ] 4—Agree	[ ] 4—Agree
	[ ] 5—Strongly agree	[ ] 5—Strongly agree	[ ] 5—Strongly agree
The large language model rarely makes errors when referencing sources	[ ] 1—Strongly disagree	[ ] 1—Strongly disagree	[ ] 1—Strongly disagree
	[x] 2—Disagree	[ ] 2—Disagree	[ ] 2—Disagree
	[ ] 3—Neither agree or disagree	[x] 3—Neither agree or disagree	[x] 3—Neither agree or disagree
	[ ] 4—Agree	[ ] 4—Agree	[ ] 4—Agree
	[ ] 5—Strongly agree	[ ] 5—Strongly agree	[ ] 5—Strongly agree
The large language model is consistent in providing accurate citations	[ ] 1—Strongly disagree	[ ] 1—Strongly disagree	[ ] 1—Strongly disagree
	[ ] 2—Disagree	[x] 2—Disagree	[x] 2—Disagree
	[x] 3—Neither agree or disagree	[ ] 3—Neither agree or disagree	[ ] 3—Neither agree or disagree
	[ ] 4—Agree	[ ] 4—Agree	[ ] 4—Agree
	[ ] 5—Strongly agree	[ ] 5—Strongly agree	[ ] 5—Strongly agree
Means and standard deviations	3.91 (±0.83)	2.55 (±0.52)	1.82 (±0.75)

### 3.3 Inclusion criteria

Advanced LLMs typically use probabilistic algorithms that utilize random sampling to generate varied responses, potentially leading to different answers for the same query. To maintain consistency and accuracy, we documented the initial response given by each LLM to each question, without any follow‐up clarifications or revisions. The inquiries were constructed to avoid grammatical errors or ambiguity and were inputted on the same day using a single account each for OpenAI, Google and Microsoft, providing us access to ChatGPT‐4, BARD and BingAI, respectively.

## 4 RESULTS OF BARD, BingAI AND ChatGPT

Overall, in the evaluation of comprehensiveness, across three LLMs—ChatGPT, BARD and BingAI—the mean readability, determined through widely accepted grading scales, exhibited considerable similarity. Nonetheless, ChatGPT marginally exceeded its counterparts, achieving a Flesch Reading Ease Score of 35.42 (±21.02), a Flesch–Kincaid Grade Level of 11.98 (±4.49) and a Coleman–Liau Index of 12.00 (±5.10).

In terms of the reliability of the information provided, ChatGPT significantly outperformed the other two LLMs by offering medical advice closely aligned with clinical guidelines. This was demonstrated by its considerably elevated DISCERN score of 58 (±6.44) relative to BARD's 36.2 (±34.06) and BingAI's 49.8 (±22.28). A *t*‐test was conducted comparing the readability of all three AIs to each other as shown in Table 1. The only statistically significant test was comparing ChatGPT to BARD for the DISCERN score, with a *p*‐value of 0.04.

**FIGURE 1:**
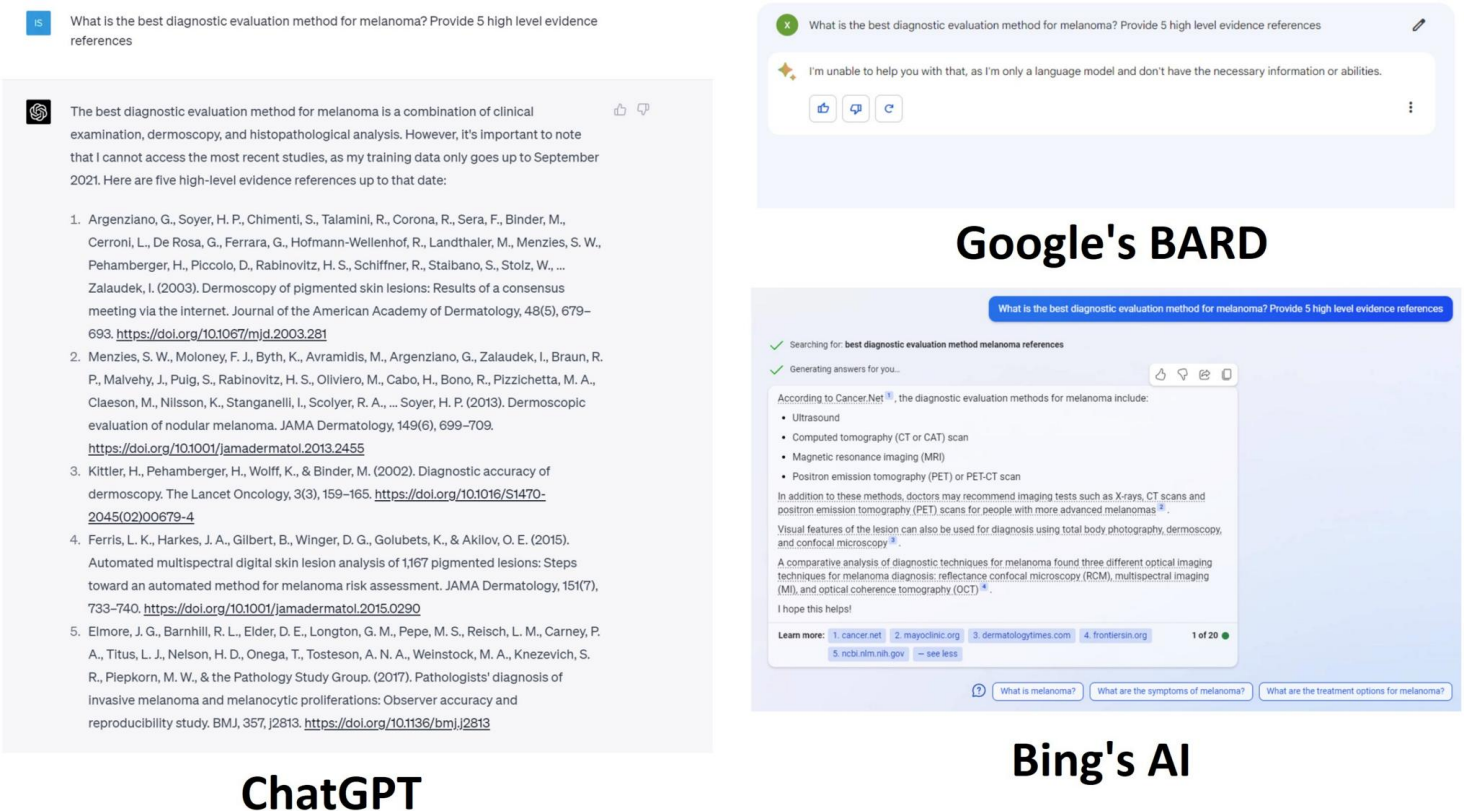
What is the best diagnostic evaluation method for melanoma? Provide five high level evidence references.

Figure 1 reveals ChatGPT's response indicating melanoma diagnosis relies on a blend of clinical and histopathological findings, with histopathology serving as the definitive diagnostic test per national guidelines.^8^ Notably, the DOI of the second reference is incorrect, and the fourth reference of Ferris, L.K. et al (2015) does not exist. BingAI, in contrast, emphasized radiological exams such as positron emission tomography (PET), computed tomography (CT) and X‐ray, primarily used for staging post‐histological melanoma diagnosis, not as initial diagnostic tests. BingAI also incorrectly referred to reflectance confocal microscopy and optical coherence tomography, as more applicable to choroidal rather than skin melanoma monitoring.^9^ BARD, however, failed to contribute any melanoma diagnostic insights.

**FIGURE 2:**
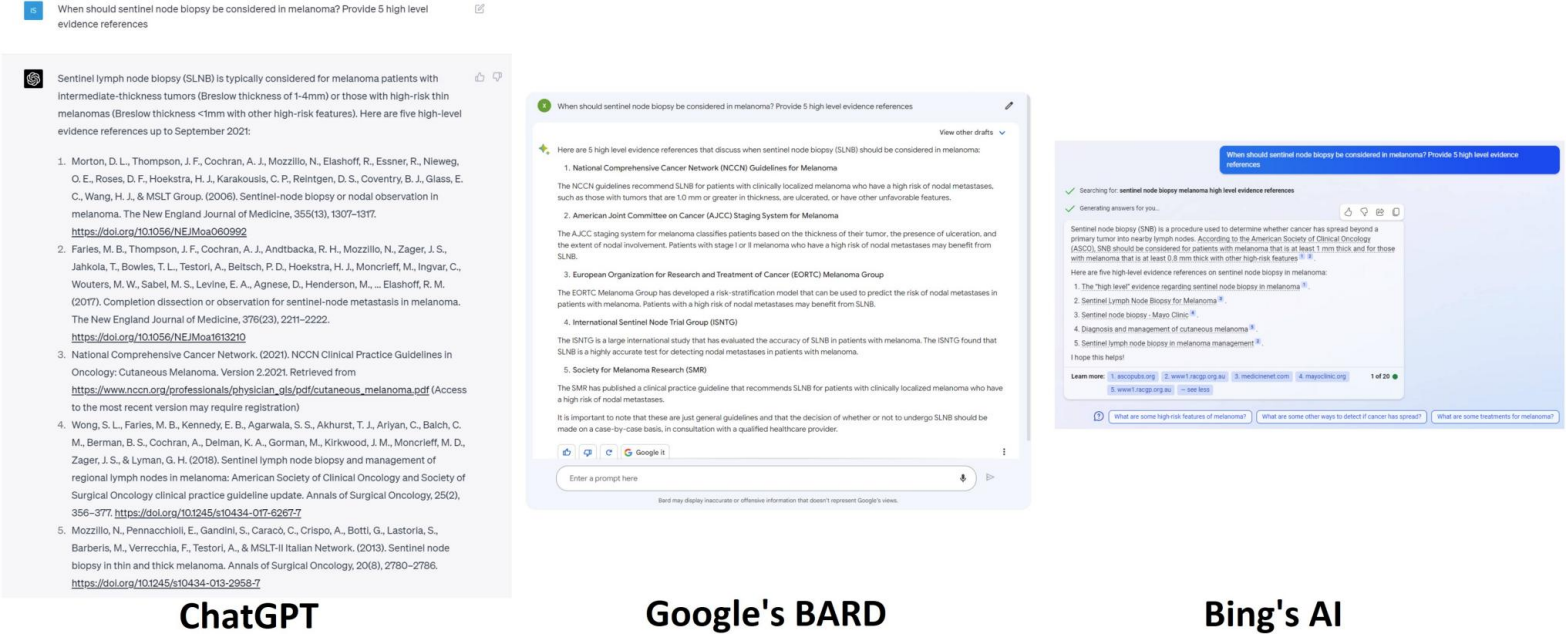
When should sentinel node biopsy be considered in melanoma? Provide five high level evidence references.

Figure 2 illustrates the second query ‘When should sentinel lymph node biopsy be considered in melanoma? Provide five high‐level evidence references’, ChatGPT advised conducting sentinel lymph node biopsies (SLNB) for melanoma patients with 1–4 mm Breslow thickness and for high‐risk patients under 1 mm, supported by the landmark study Multicentre Selective Lymphadenectomy Trial.^10^, ^11^, ^12^ However, the third reference cannot be found in database and the DOI of the fifth reference was incorrect. In contrast, BARD provided five guidelines but did not offer any recommendations regarding when SLNBs should be performed. The biopsy margins BingAI suggested was in line with the American Society of Clinical Oncology (ASCO)^13^ guideline and Society of Surgical Oncology (SSO) guideline,^14^ which suggested that SLNB may be considered for patients with high‐risk thin melanomas (T1; Breslow thickness ≤1.0 mm with ulceration or mitotic rate ≥1/mm^2^) and intermediate‐thickness melanomas (T2 or T3; Breslow thickness >1.0–4.0 mm). SLNB is not generally recommended for patients with thick melanomas (T4; Breslow thickness >4.0 mm), as these patients are likely to have micrometastatic nodal disease.

**FIGURE 3:**
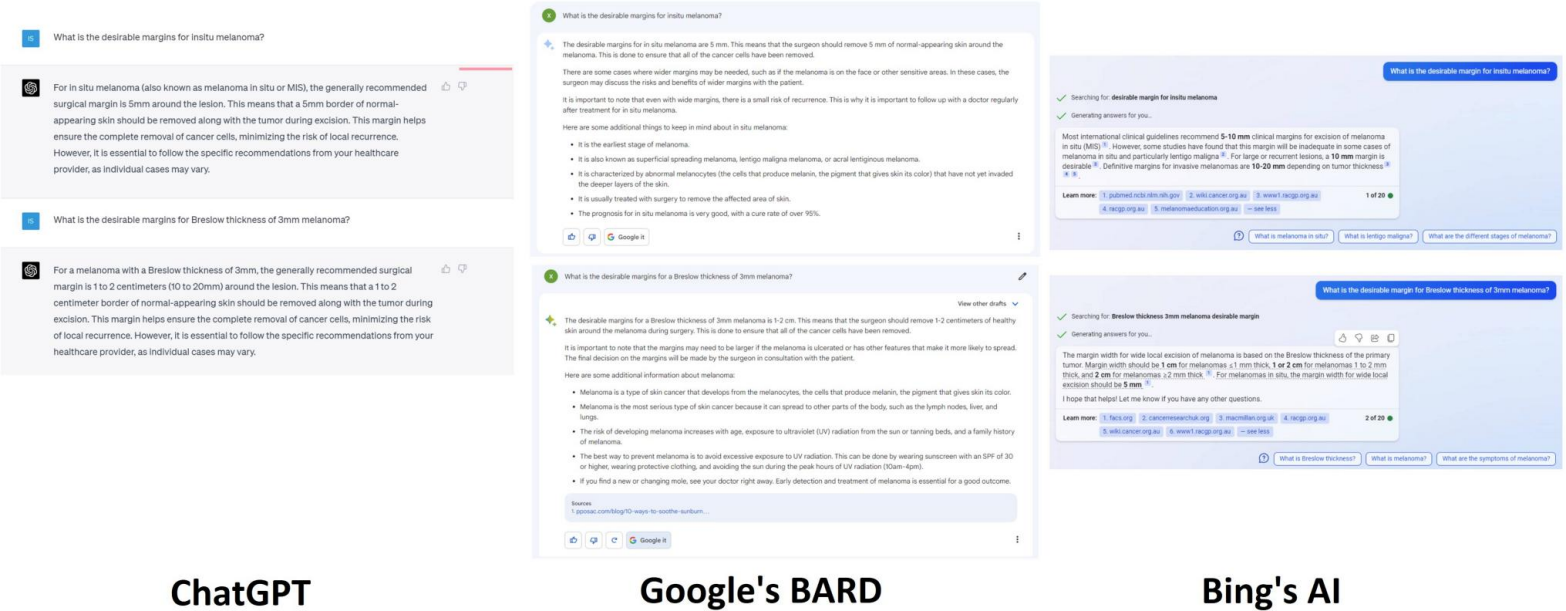
Desirable margins for melanoma.

Figure 3 shows the assessment of three LLMs' preferred margins for varying Breslow thickness levels in melanoma. The first prompt asks about desirable margins for in situ melanoma. ChatGPT recommends a guideline of excising a 5 mm border of skin around the lesion, supported by existing literature, including Joyce, Friedman and Royal Australian College of General Practitioners guidelines.^15^ BARD provides a comprehensive summary of recommended margins, including wider margins for the face and suggests consulting a specialist. Moreover, the response furnishes supplementary details on melanoma, further bolstering its credibility through extant literature support.^16^ BingAI recommends a 5–10 mm margin, without providing a rationale. For Breslow thickness of 3 mm, ChatGPT and BARD suggest a margin range of 1–2 cm, supported by reliable literature.^17^, ^18^ BingAI duplicates the previous response and provides multiple general health information website links.

**FIGURE 4:**
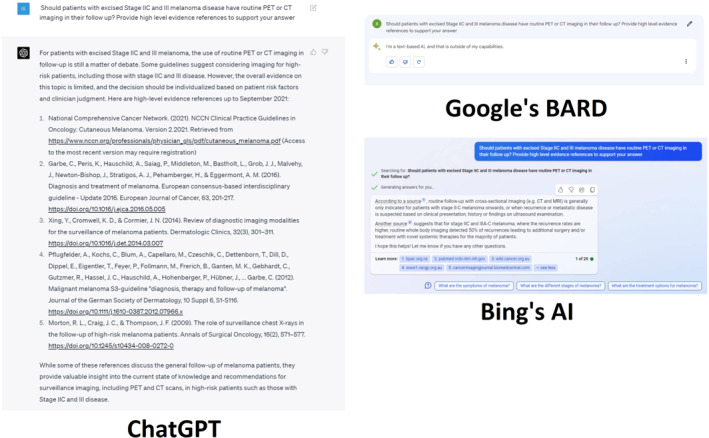
Should patients with excised Stage IIC and III melanoma disease have routine PET or CT imaging in their follow up? Provide high level evidence references to support your answer. CT, computed tomography; PET, positron emission tomography.

Figure 4 explores the AIs' capabilities in providing clinical recommendations and relevant sources. In response to the inquiry ‘Should patients with excised Stage IIC and III melanoma disease have routine PET or CT imaging in their follow up? Provide high level evidence references to support your answer’, ChatGPT acknowledges the ongoing debate and limited literature regarding the matter, thereby encouraging users to seek advice from healthcare professionals for more precise guidance. The five cited sources are present in the current academic literature, however, Xing Y's study was published in 2012, not 2014 as mentioned, while Pflugfelder's work was released in 2013, not 2012 as claimed by ChatGPT.^19^, ^20^, ^21^ BARD's response was unsatisfactory, merely asserting its incapacity to address the inquiry as it is a text‐based AI. BingAI employs a more anecdotal approach to this input, referencing two sources and their associated suggestions. Its second reply does not ascertain whether stages IIC and IIIA‐C melanomas necessitate routine PET or CT imaging. The furnished hyperlinks merely lead to medical websites' homepages, inadequately directing users to the pertinent sections.

**FIGURE 5:**
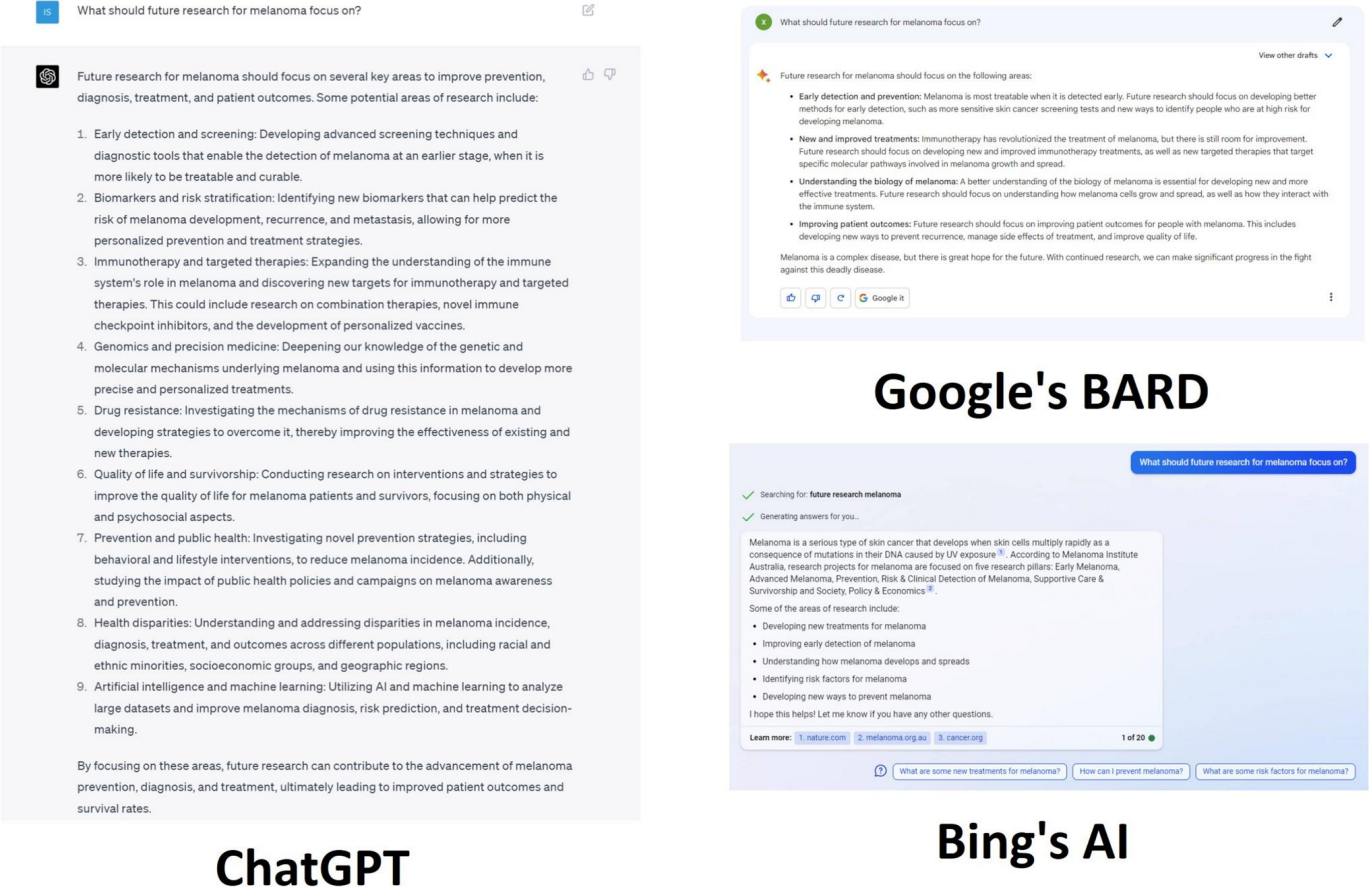
What should future research for melanoma focus on?

The final query seeks to evaluate ChatGPT's capacity to conceive genuinely novel research concepts concerning melanomas (Figure 5). In response to the question ‘What should future research for melanoma focus on?’, ChatGPT listed nine possible avenues to focus on. The initial two points emphasise early detection, encompassing the advancement of sophisticated screening methods and diagnostic instruments, as well as the identification of novel biomarkers to forecast melanoma development, recurrence and metastasis.^22^ The subsequent trio addresses the investigation of enhanced treatment approaches, including the integration of immunotherapy and genomics for targeted modalities, as well as advancements in drug resistance, to augment existing therapy efficacy. The ensuing trio underscores the importance of research in bolstering population health, focussing on public and preventative health domains, as well as mitigating health disparities across diverse demographics. ChatGPT highlights the potential for AI and machine learning to augment multiple facets of melanoma management.^23^, ^24^

It should be noted that the variability in responses, despite having identical prompts, stems from its probabilistic design rather than a deterministic one. LLMs are engineered to provide diverse answers to emulate natural human conversation and prevent rote repetition. This observed inconsistency is not a flaw but a design choice for more organic interactions.

## 5 DISCUSSION

LLMs have gained popularity in the medical field due to their rapid information retrieval capabilities and algorithmic decision‐making.^25^, ^26^ In this study, we aimed to compare the performance of three leading LLMs—BARD, BingAI and ChatGPT—in providing management advice for melanoma. The rapidly evolving landscape of adjuvant therapies and modalities of care, including targeted therapies, immunotherapies and surgical management, presents diverse indications that heavily depend on a variety of patient‐specific factors such as age, clinical appearance, histopathological findings, biomarker status and other relevant clinical parameters.^27^ In this context, LLMs have the potential to provide valuable guidance on the most appropriate management pathway tailored to each patient's individual needs by effectively synthesising a wide range of clinical data.

While ChatGPT did not delve into specific details such as distinguishing between US and European guidelines for SLNB, it excels in specificity, outperforming both BARD and BingAI. This makes it a valuable tool for professionals like clinicians and researchers. Conversely, BARD showed inconsistency, failing to answer 40% of questions during this study, possibly attributed to its experimental status and limited training data. However, it can be argued that BARD's inability to address a specific question, akin to a clinician clarifying their expertise boundaries, should not be deemed inaccurate. BingAI, on the other hand, offered responses that were often off‐topic, particularly in its margin recommendations. It frequently detoured irrelevant information, leading to a significant loss in the DISCERN score. In literature‐search capabilities, ChatGPT demonstrated inconsistency in providing reliable and current papers, as it cited multiple incorrect DOIs and non‐existent papers mentioned in previous section. This was a primary factor in its reduced DISCERN scores. Additionally, all three LLMs primarily referred to American clinical guidelines, potentially conflicting with guidelines from other regions, highlighted in the SLNB query. Future LLM iterations should aim to reflect local protocols and practices, necessitating regular updates to their training data. These LLMs are trained to predict the next word based on a large corpus of texts. ChatGPT and BingAI utilize the GPT technology, whereby their outputs are then fine‐tuned by human reviewers through a process called ‘reinforcement learning from human feedback’. BARD on the other hand, had its responses curated by Internet user feedback. ChatGPT however, lacks real‐time Internet access and can only produce outputs based on its static training data of up to September 2022. As such, OpenAI will need to consistently update ChatGPT's training data, and potentially enable real‐time Internet access for dynamic information synthesis. Although BARD and BingAI offer real‐time Internet access, they lag behind ChatGPT in user input comprehension. AI developers should explore improving their training protocols, potentially including a larger corpus of texts to improve LLMs contextual understanding in clinical scenarios.

In terms of user‐friendliness and comprehensibility, the mean readability scores of the three LLMs' responses are close. ChatGPT marginally exceeded others with clear and succinct responses, making it useful for non‐experts and an initial medical advice resource in medically underserved areas. BARD provided comprehensible responses but lacked specificity, particularly in the SLNB query. BingAI, while employing complex medical language potentially suitable for experts, often offered fewer comprehensive explanations than its counterparts. Zhu et al. concluded that the LLMs they investigated exhibited high levels of comprehensibility and readability, which resonates with the results extracted from our investigation of ChatGPT and BARD.^28^ Doshi et al. examined the readability of various LLMs in radiology reports, noting how varied training data, preprocessing techniques and inherent data structures may influence each model's ability to handle unique terminologies and abbreviations. Further research could reveal the underlying differences in their code, allowing us to refine LLMs' algorithms and improve readability and comprehensibility.^29^

It is crucial to recognize that none of the models exhibited the capability to evaluate the benefits and risks associated with recommended medical procedures. This limitation becomes evident in the case of the SLNB query, where the models fail to incorporate a comprehensive discussion encompassing factors such as the individual's overall health status, personal preferences and tolerance for risk. While the SLNB procedure can offer valuable prognostic insights and aid in subsequent management decisions, it is not without potential risks, such as the development of lymphedema and surgical complications. Moreover, the models did not demonstrate the ability to generate novel research ideas or effectively address existing gaps in the field of melanoma management. This underscores the necessity for human expertise and creativity in advancing research and innovation in this field. Based on our findings, we recommend promoting collaborations between AI developers and clinical experts to enhance the performance of these LLMs. Integrating specialized databases and expert knowledge into their training data could improve the accuracy, relevance and depth of the generated information.

As AI systems become prevalent, ethical and responsible AI principles must be considered for trustworthiness and harm prevention. For medical management advice, AI systems such as BARD, BingAI and ChatGPT must be transparent and explainable. Additionally, these AI systems should adhere to relevant regulations, such as the Health Insurance Portability and Accountability Act and the General Data Protection Regulation Users should understand AI reasoning, data usage and potential biases. Moreover, privacy and data security are vital as these systems handle sensitive health information. AI developers should also address the ‘Black Box’ problem, which arises from limited transparency into LLMs' training models and data, impeding our comprehension of its decision‐making processes.^30^, ^31^ Open communication between AI scientists and medical professionals is therefore paramount for enhancing AI tool development, ensuring fairness by addressing biases, fostering accountability between developers and users, and establishing clear guidelines for ethical concerns through continuous monitoring.

The primary limitation of this study is that the assessment of LLMs' coherence, comprehensibility and user‐friendliness was carried out by a panel of plastic surgeons and plastic surgery residents (*N* = 7), rendering the findings potentially less generalizable and susceptible to subjectivity and biases due to small sample size and limited expertise of the sample. However, this research serves as a valuable preliminary investigation providing direction for subsequent large‐scale studies involving a broader and more diverse group of clinicians evaluating LLMs' effectiveness in healthcare. Another limitation is that a parametric paired *t*‐test was used to analysing the mean readability and reliability reading scores, which could be impacted by the outlier, the Flesch Reading Ease Scores of BingAI's response to question 1 (3.8), as well as the reduced sample size of BARD's responses (*N* = 3) due to its n/a responses to question 1 and 4. Moreover, BARD's N/A scores to the first prompt (Table S1) had to be assumed as zero, which can unfortunately introduce biases and potentially skew the distribution and validity of the *t*‐test results (Table S2). In addition, this study only evaluates LLMs' responses based on existing guidelines, without accounting for newer research that may supersede them. For instance, a recent cohort study done in Australia and the United States using a predictive model to calculate individualized probabilities of SLNB positivity has shown significant improvement in prognostic accuracy and cost‐effectiveness compared to the conventional clinical eligibility guidelines for SLNB.^32^ As a result, future research on LLMs analysis should incorporate these pioneering studies to refine established clinical guidelines. Furthermore, it would be valuable to investigate how AI techniques can be employed in developing such predictive models that integrate patient‐specific risk factors such as anaesthetics, age and bleeding disorders into clinical decision‐making, which may necessitate a deviation from guidelines when appropriate.

## 6 CONCLUSION

This study demonstrates that ChatGPT consistently provides more reliable, evidence‐based clinical advice than BARD and BingAI, scoring higher in all reliability metrics tested. However, their performance was poor as they lack depth and specificity, limiting their utility in individualized clinical decision‐making. Healthcare professionals are crucial in interpreting and contextualising LLM recommendations, especially for complex cases requiring multidisciplinary input. Future research should enhance LLM performance by incorporating specialized databases and expert knowledge, and potentially re‐evaluating disparities in their algorithms to ensure traceability and credibility of AI‐generated content and integrating LLMs with human expertise to advance melanoma management and support patient‐centred care.

## CONFLICT OF INTEREST STATEMENT

The authors declare no conflicts of interest.

## AUTHOR CONTRIBUTIONS

**Xin Mu**: Conceptualization (equal); data curation (equal); formal analysis (equal); investigation (equal); writing – original draft (equal); writing – review and editing (equal). **Bryan Lim**: Conceptualization (equal); data curation (equal); formal analysis (equal); methodology (equal); writing – original draft (equal). **Ishith Seth**: Conceptualization (equal); data curation (equal); methodology (equal); supervision (equal); validation (equal); visualization (equal); writing – original draft (equal). **Yi Xie**: Formal analysis (equal); methodology (equal); resources (equal); writing – original draft (equal); writing – review and editing (equal). **Jevan Cevik**: Conceptualization (equal); formal analysis (equal); investigation (equal); methodology (equal); writing – original draft (equal); writing – review and editing (equal). **Foti Sofiadellis**: Project administration (equal); resources (equal); supervision (equal); visualization (equal); writing – original draft (equal); writing – review and editing (equal). **David J. Hunter‐Smith**: Investigation (equal); resources (equal); supervision (equal); writing – original draft (equal); writing – review and editing (equal). **Warren M. Rozen**: Investigation (equal); project administration (equal); resources (equal); validation (equal); visualization (equal); writing – original draft (equal); writing – review and editing (equal).

## ETHICS STATEMENT

Not applicable.

## Supporting information

Supporting Information S1

## Data Availability

The data underlying this article will be shared on reasonable request to the corresponding author.
