# Supplementary material for: Comparison of large language models in management advice for melanoma: Google's AI BARD, BingAI and ChatGPT
Source: Skin Health Dis. 2023 Nov 28;4(1):e313. doi: 10.1002/ski2.313 (PMC10831541; doi:10.1002/ski2.313)
Supplement: Supplementary file 1 — Supporting Information S1 [file SKI2-4-e313-s001.docx]

**Supplementary Table 1.** Mean readability and reliability of LLMs' responses

|  |  | Readability | | | Reliability |
| --- | --- | --- | --- | --- | --- |
|  | Prompts | Flesch Reading Ease Score | Flesch-Kincaid Grade Level | The Coleman-Liau Index | DISCERN score |
| **ChatGPT** | 1. Diagnostic methods of melanoma | 46.9 | 7.4 | 8 | 60 |
|  | 2. SLNB | 17 | 18.1 | 16 | 59 |
|  | 3. Desirable margins | 64.2 | 8 | 6 | 75 |
|  | 4. Routine imaging follow up | 35.2 | 11.9 | 12 | 70 |
|  | 5. Future research | 13.8 | 14.5 | 18 | 64 |
| Mean(sd) |  | 35.42 (21.02) | 11.98 (4.49) | 12.00 (5.10) | 65.6 (6.80) |

|  |  | Readability | | | Reliability |
| --- | --- | --- | --- | --- | --- |
|  | Prompts | Flesch Reading Ease Score | Flesch-Kincaid Grade Level | The Coleman-Liau Index | DISCERN score |
| **BARD** | 1. Diagnostic methods of melanoma | N/A | N/A | N/A | 0 |
|  | 2. SLNB | 20.2 | 17.7 | 13 | 50 |
|  | 3. Desirable margins | 53.5 | 11.2 | 8 | 73 |
|  | 4. Routine imaging follow up | N/A | N/A | N/A | 0 |
|  | 5. Future research | 22.6 | 16.2 | 14 | 58 |
| Mean(sd) |  | 32.1 (18.57) | 15.03 (3.40) | 11.67 (3.21) | 36.2 (34.06) |

|  |  | Readability | | | Reliability |
| --- | --- | --- | --- | --- | --- |
|  | Prompts | Flesch Reading Ease Score | Flesch-Kincaid Grade Level | The Coleman-Liau Index | DISCERN score |
| **BingAI** | 1. Diagnostic methods of melanoma | 3.8 | 20 | 15 | 12 |
|  | 2. SLNB | 43.1 | 13.3 | 10 | 72 |
|  | 3. Desirable margins | 34.9 | 13 | 12 | 70 |
|  | 4. Routine imaging follow up | 44.7 | 8.1 | 12 | 52 |
|  | 5. Future research | 22.9 | 13.5 | 14 | 61 |
| Mean(sd) |  | 29.88 (16.94) | 13.58 (4.23) | 12.6 (1.95) | 53.4 (23.7) |

**Supplementary Table 2.**T-test difference analysis of LLM’s readability and reliability

|  | ChatGPT versus BARD | ChatGPT versus BingAI | BARD versus BingAI |
| --- | --- | --- | --- |
| Flesch Reading Ease Score | 0.8297 | 0.6585 | 0.8678 |
| Flesch-Kincaid Grade Level | 0.2758 | 0.5780 | 0.6345 |
| The Coleman-Liau Index | 0.9235 | 0.8120 | 0.6199 |
| DISCERN score | 0.0461 | 0.0918 | **0.4147** |
